# Minimal Invasive Radioguided Ectopic Parathyroidectomy in Upper Mediastinum

**DOI:** 10.4274/mirt.galenos.2019.09709

**Published:** 2019-09-06

**Authors:** Zehra Pınar Koç, Turgut Karlıdağ, Pelin Özcan Kara, Abdulvahap Akyiğit, Ferda Dağlı

**Affiliations:** 1 Mersin University Faculty of Medicine, Department of Nuclear Medicine, Mersin, Turkey; 2 Fırat University Faculty of Medicine, Department of Ear Nose and Throat, Elazığ, Turkey; 3 Elazığ Training and Research Hospital, Clinic of Ear, Nose and Throat, Elazığ, Turkey; 4 Fırat University Faculty of Medicine, Department of Pathology, Elazığ, Turkey

**Keywords:** Hyperparathyroidism, adenoma, minimally invasive, radioimmunodetection

## Abstract

In this study we wanted to present a case with the history of multiple previous neck explorations and persisting upper mediastinal ectopic parathyroid adenoma who underwent a successful operation with radioguided minimal invasive approach.

## Abstract (tr)

Bu çalışmada çok sayıda boyun operasyonu öyküsü ve kalıntı üst mediastinal ektopik paratiroid adenomu olan ve başarılı bir şekilde radyoişaretli minimal invaziv yaklaşımla opere edilen bir olguyu sunmak istedik.

## Figures and Tables

**Figure 1:**
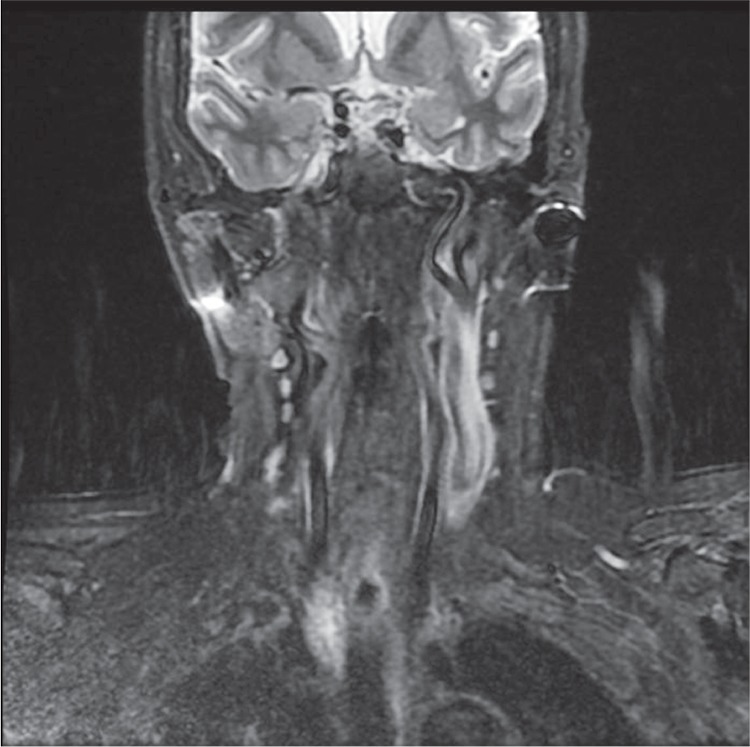
The magnetic resonance images showed remaining upper mediastinal parathyroid adenoma

**Figure 2:**
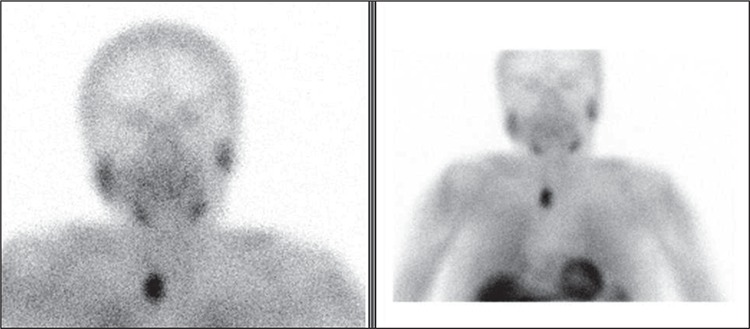
Anteroposterior early phase Tc-99m MIBI parathyroid planar image including the neck and mediastinum showing right upper mediastinal increased uptake consisted with the ectopic parathyroid adenoma. A 69-year-old female patient with the diagnosis of hyperparathyroidism; hypercalcemia and increased parathyroid hormone (PTH) levels and additional multinodular goiter was referred to our department for labeling for the radioguided surgery. The patient had anamnesis of bilateral total thyroidectomy due to multinodular goiter and an additional failed surgery for the parathyroid adenoma excision due to the ectopic localization of adenoma. She had previous diagnosis of incidental papillary microcarcinoma of the thyroid gland. Persistent hipercalcemia and hyperparathyroidism were observed after the two operative procedures. Parathyroid scintigraphy was performed by intravenous injection of 370 mBq Tc-99m MIBI with low energy high resolution collimator equipped double head SPECT gamma camera prior to the surgery. The clear demonstration of a parathyroid adenoma in planar images obviated the need for SPECT imaging. The patient was referred to the operation after scintigraphy imaging for radioguided surgery with handheld gamma probe. In the operation room, the background count per minute (cpm) level was 5 from the shoulder and postoperative field count was 200 and the count of the lesion was 1500 cpm. The lesion was between the trachea and esophagus (retroesophageal) and the surgery was performed by a small incision of 2 cm wide. The lesion which was confirmed as a parathyroid adenoma by the pathology result was 2,704 gram in weight and 3 x 1.6 x 0.8 cm in size. Successful removal was confirmed both by postoperative counts and quick PTH analysis (preoperative: 698 pg/mg postoperative: 70.9 pg/mg (normal values: PTH 14-74 pg/mg). No complication was observed during or after the surgery. The patient was normocalcemic after six months of follow up. The definition of minimal invasive parathyroidectomy includes focused surgery planned for the excision of a single enlarged parathyroid gland (1). Routine parathyroid surgery includes inspection and exploration of all the neck, however in recent years the dissemination of imaging methods has contributed to development of this novel surgical approach (minimal invasive surgery) which is a targeted and secure way of successful surgery. Minimally invasive procedures are more comfortable for both the surgeon and the patient due to the cost effectiveness, being safe in terms of complications, and reduced operation, recovery and hospitalization times. Ectopic parathyroid adenoma is a diagnostic challenge which may be located in anywhere between angle of mandibule and mediastinum (2). Approximately 70% of the patients that have failed surgery are due to the ectopic localization of the parathyroid adenoma (3). Most of the upper mediastinal lesions might be excised by a cervical incision (4). However, preoperative localization methods are necessary for successful result (5). Currently the most accurate and reliable method for the localization of the parathyroid adenoma is the parathyroid scintigraphy with additional ultrasonography. Planar parathyroid scintigraphy achieves a sensitivity of 78% and with additional SPECT imaging this ratio increases to 96% according to the previous reports (6). There is limited number of reports of the radioguided surgery for mediastinal lesions. In a previous report, Doğan et al. (7) considered gamma probe as a useful method for ectopic parathyroid adenoma operation. Also in a previous series involving minimal invasive procedures of 102 patients, radioguided surgery was found to be an efficient method, especially in the upper mediastinal lesions (8). The reported patient in this study had a successful surgery and excellent outcome after her third operation without any complications. This case reports shows that minimal invasive radioguided surgery is also possible and required for the patients with history of multiple previous neck explorations and upper mediastinal lesions.
